# Reliability of the Acoustic Voice Quality Index AVQI and the Acoustic Breathiness Index (ABI) when wearing CoViD-19 protective masks

**DOI:** 10.1007/s00405-022-07417-4

**Published:** 2022-05-06

**Authors:** Bernhard Lehnert, Jeffrey Herold, Markus Blaurock, Chia-Jung Busch

**Affiliations:** Department of Oto-Rhino-Laryngology, Phoniatrics and Pedaudiology Division, University Medicine Greifswald, Greifswald University, Sauerbrauchstraße, 17453 Greifswald, Germany

**Keywords:** Dysphonia, Voice examination, AVQI, ABI, CoViD-19 protective mask

## Abstract

**Purpose:**

Investigating whether the Acoustic Voice Quality Index (AVQI) and the Acoustic Breathiness Index (ABI) are valid and comparable to previous unmasked measurements if the speaker wears a surgical mask or a FFP-2 mask to reduce the risk of transmitting air-borne viruses such as SARS-CoV-2.

**Methods:**

A convenience sample of 31 subjectively healthy participants was subjected to AVQI and ABI voice examination four times: Twice wearing no mask, once with a surgical mask and once with a FFP-2 mask as used regularly in our hospital. The order of the four mask conditions was randomized. The difference in the results between the two recordings without a mask was then compared to the differences between the recordings with each mask and one recording without a mask.

**Results:**

Sixty-two percent of the AVQI readings without a mask represented perfectly healthy voices, the largest AVQI without a mask value was 4.0. The mean absolute difference in AVQI was 0.45 between the measurements without masks, 0.48 between no mask and surgical mask and 0.51 between no mask and FFP-2 mask. The results were neither clinically nor statistically significant. For the ABI the resulting absolute differences (in the same order) were 0.48, 0.69 and 0.56, again neither clinically nor statistically different.

**Conclusion:**

Based on a convenience sample of healthy or only mildly impaired voices wearing CoViD-19 protective masks does not substantially impair the results of either AVQI or ABI results.

## Introduction

In the years 2020 and 2021 the worldwide Corona Virus Disease 2019 (CoViD-19) epidemic has changed social interactions and the way medicine is practiced. In 2020 the Union of the European Phoniatricians (UEP) published a “Position Statement relating to Phoniatric and Laryngological services during the COVID-19 pandemic” in which personal protective equipment such as FFP3 masks is advised “even if the patient is totally asymptomatic as long as the procedures include examination or manipulation of the patient’s throat, nose, larynx or upper airway” [1]. It advised to postpone surgery and to assess and treat speech, language and voice disorders wholly in a remote manner, utilizing teletherapy, whenever possible. However, in some countries, regulations made teletherapy difficult. Asadi et al. 2019 emphasized that not only coughing and breathing but also normal speech yields large quantities of particles that are large enough to carry a variety of communicable respiratory pathogens [2]. They demonstrated, that the rate of particle emission during normal human speech increased with the loudness. A number of so-called superspreader events were associated with people singing, such as described in a Centers for Disease Control and Prevention (CDC) report of 32 confirmed and 20 probable secondary CoViD-19 cases after one 2.5 h long choir practice attended by 61 people [3].

All of this raises the question of best practices in voice examination in the ongoing CoViD-19 epidemic. Medical masks worn by our patients protect medical staff. But during voice examination they may hinder jaw motions, especially mouth opening, they may impede breathing air resistance and the subjective feel of breathing freely and they may muffle the sound. It is therefore important to quantify those influences on our results. Whilst this study is centered around two specific objective measures of voice quality, a variety of measurements has already been investigated with regards to CoViD-19 protective mask use:

Cavallaro et al. did not detect differences in maximum phonation time, frequency, jitter, shimmer and harmonics-to-noise ratio due to surgical masks [4]. Fiorella et al. did not detect differences in numerous voice measurements in male as well as female participants when wearing masks [5]. Joshi et al. investigated the influence of a number of different mask types on intensity, fundamental frequency, smoothed cepstral peak prominence, first and second formant frequency and found no substantial difference when not wearing a mask [6]. In a study by Lin et al., healthy participants showed a significantly higher SPL, a smaller perturbation and a decrease in F3 when wearing medical masks [7]. Gojayev et al. looked at the frequency, Jitter, Shimmer, harmonics-to-noise-ratio, *s*/*z* ratio and maximum phonation time with surgical as well as valved FFP-3 masks. Their study stands out for a comparably large number of 104 included patients. Despite the consequent statistical power, in the comparison of “no mask” to “surgical mask” none of their comparison gained statistical significance, except one paper which noticed significant differences in shimmer and harmonic to noise ratio with FFP3" [8].

The Acoustic Voice Quality Index (AVQI) and the Acoustic Breathiness Index (ABI) are computer-based algorithms to judge voice quality and determine hoarseness (in AVQI) and breathiness (in ABI) [9–11]. They are based on a recording of a sustained vowel /a/ and a short reading passage, so no increased loudness is needed and it has been validated in a large number of languages, amongst them German [12], which is the language this study was conducted in. From those voice recordings, each index is computed as an objective measure of voice quality. Each is represented by a value, usually between 0 and 10, with smaller numbers indicating healthier voices. The AVQI as a measure of hoarseness corresponds to the G, ABI as a measure of breathiness to the B, in the well-known GRBAS scale [13]. AVQI values below 1.85 are predictive of a G0 rating in German, ABI values below 3.42 are predictive of a B0 rating in German [12].

In the course of the pandemic, masks on nose and mouth were increasingly considered one of the main means to fight virus transmission. In our primary care medical facility, staff and students were mandated to carry Filtering Face Pieces grade 2 according to European Norm EN 149 (in short: FFP-2 masks) regularly in patient contact, patients were asked to wear surgical masks wherever and whenever feasible.

This study’s aim was to investigate, whether the use of surgical or FFP-2 masks during AVQI and ABI measurement leads to biased or unbiased results and so whether AVQI and ABI values can be reliably measured with patients wearing masks.

## Material and methods

Thirty-one healthy medical and logopedics students volunteered to participate in this study.

Each participant underwent AVQI and ABI measurement four times: twice without a mask, once with a surgical mask and once with an FFP-2 mask. The order of the measurements was randomized according to a predefined randomization plan. The predefined plan also defined one of the measurements without a mask to be the base measurement to which all other measurements were compared. Thus, sometimes the measurements with a mask were compared to the first, sometimes to the second measurement without a mask, all according to a randomly predefined plan.

Voice samples were recorded using a AKG C 544L Vocal condenser microphone at 10 cm off-axis microphone-mouth-distance and a Focusrite iTrack Solo external sound card on a portable computer in a moderately sound-treated room that is ordinarily used for voice examinations in our department and usually has background noise levels below 40 dB.

The spread of the absolute differences between the two measurements without a mask was compared to the spread of absolute differences between the base measurement and with the type of mask by graphical means, descriptive statistics and via Wilcoxon’s signed-rank test. All computations were performed using R 4.1.0 [14] and the exactRankTests package [15].

The participants were exposed to no risk and no personalized data was acquired so that according to local laws and regulations an ethics committee vote was uncalled-for.

## Results

Thirteen participants identified as male (42%), 18 as female (58%). Descriptive statistics of the observed AVQI and ABI values are given in Table 1. The mean AVQI values in each of the four measurement conditions varied from 1.4 to 1.8, thus below the aforementioned G0-cutt-off at 1.85. The mean ABI values in each of the four measurement conditions varied from 2.2 to 2.8, thus below the aforementioned B0-cut-off at 3.42. It needs to be considered, that these “cut off” values come with less then 100% sensitivity and specificity and mark the transition from “normal voice” to “mild disturbance”, and not the transition from healthy to diseased.

**Tab 1:** Descriptive statistics of the observed voice indices

Index	Mask	*n*	Mean	SD	Median	Min	Max	Range	SE
AVQI	w/o (ref)	31	1.8	0.8	1.7	0.1	4.0	3.9	0.1
	w/o	31	1.6	0.9	1.7	0.0	3.8	3.8	0.2
	Surgical	31	1.5	0.9	1.5	0.0	3.2	3.2	0.2
	FFP-2	31	1.4	0.9	1.4	− 0.3	3.1	3.5	0.2
ABI	w/o (ref)	31	2.8	1.0	3.0	1.0	4.0	3.0	0.2
	w/o	31	2.7	1.2	2.9	0.6	4.9	4.3	0.2
	Surgical	31	2.5	1.2	2.9	0.2	4.9	4.7	0.2
	FFP-2	31	2.2	1.2	2.6	-0.6	3.8	4.3	0.2

The order or measurements was randomized, ‘*ref*’ is the measurement without a mask that the other three measurements were compared to. ‘*se*’ denotes the standard error of the mean

**Fig. 1:**
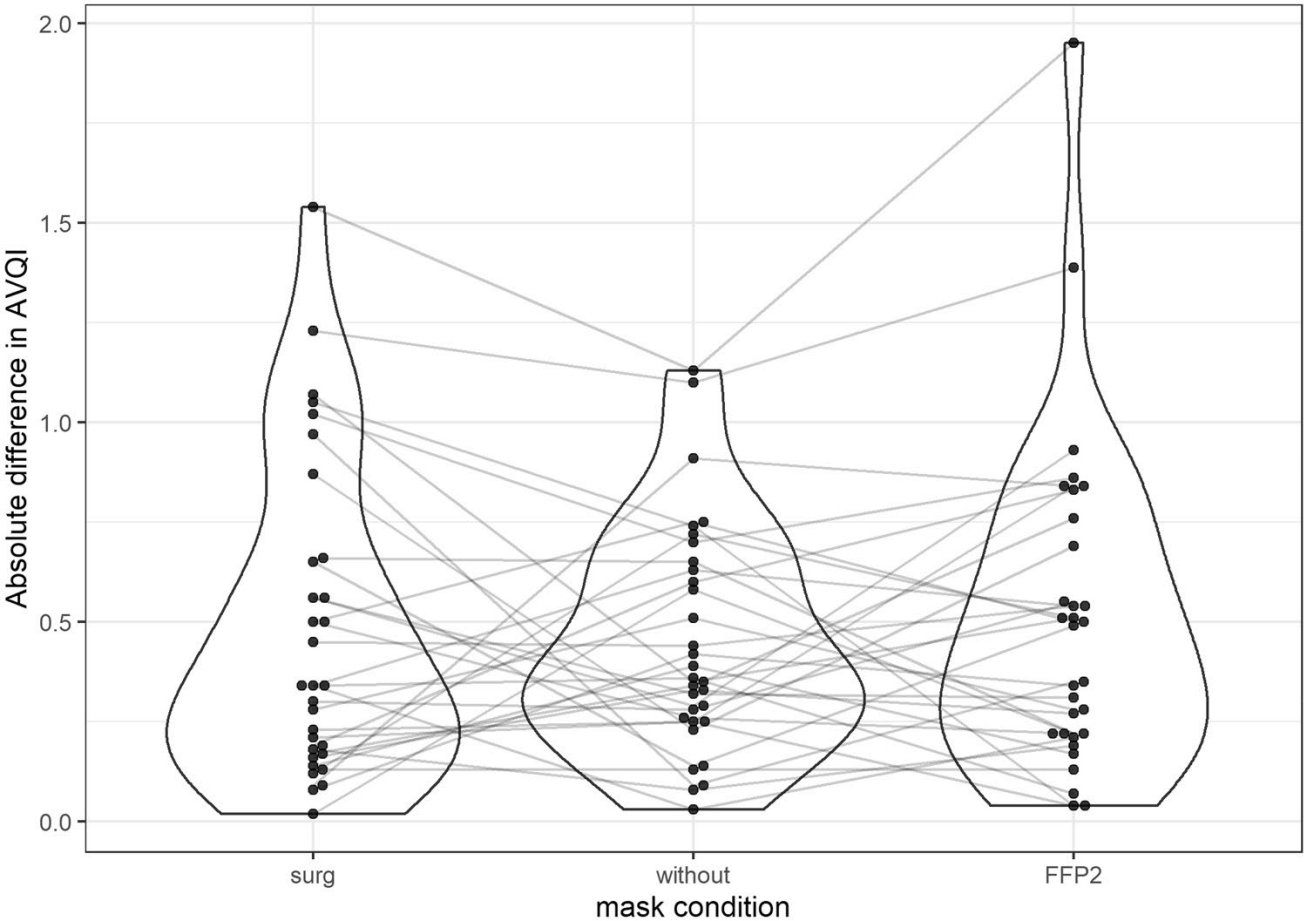
Absolute differences of the AVQI to a base measurement without a mask. The measurement without represents the normal retest variance to which the measurements with surgical and FFP-2 mask are compared

**Fig. 2:**
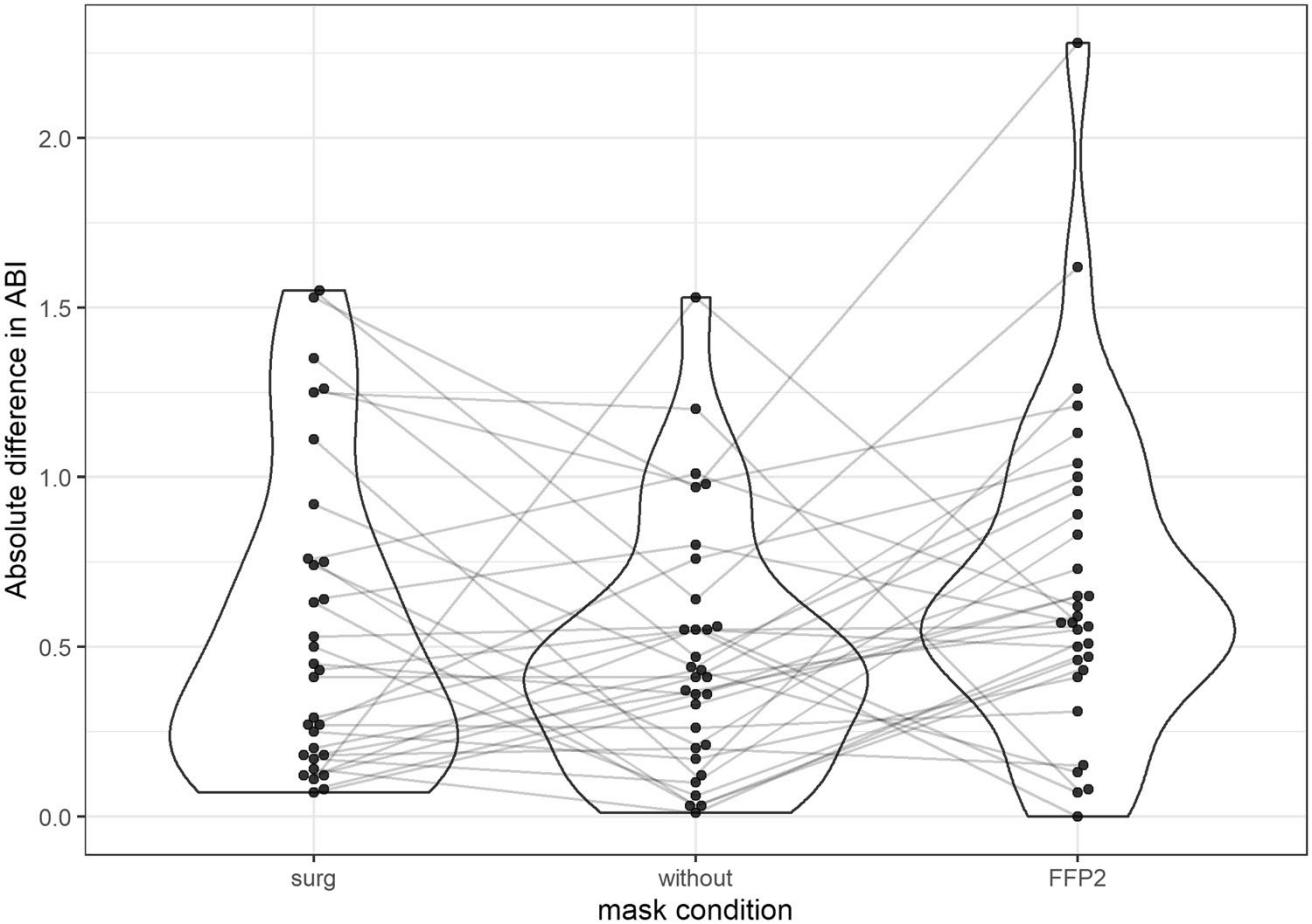
Absolute differences of the ABI to a base measurement without a mask. The measurement without represents the normal retest variance to which the measurements with surgical and FFP-2 mask are compared

The absolute differences between the base measurement (one of the two measurements without a mask that was chosen in advance) and the three comparison measurements are given in Table 2. Figure 1 depicts the AVQI differences and Fig. 2 the ABI differences.

**Tab 2:** Descriptive statistics of the calculated differences between the voice indices

Index	Mask	*n*	Mean	SD	SE	Min	Q0.1	Q0.25	Median	Q0.75	Q0.9	Max
AVQI	Without	31	0.452	0.286	0.051	0.03	0.13	0.255	0.36	0.640	0.75	1.13
	FFP2	31	0.509	0.413	0.074	0.04	0.13	0.220	0.49	0.725	0.86	1.95
	Surg	31	0.482	0.394	0.071	0.02	0.12	0.175	0.34	0.656	1.05	1.54
ABI	Without	31	0.480	0.368	0.066	0.01	0.06	0.205	0.41	0.600	0.98	1.53
	FFP2	31	0.685	0.480	0.086	0.00	0.13	0.445	0.57	0.925	1.21	2.28
	Surg	31	0.557	0.457	0.082	0.07	0.12	0.180	0.43	0.755	1.26	1.55

‘se’ denotes the standard error of the mean, ‘Q0.1’ is the 10% quantile, ‘Q0.25’ the 25% quantile and so on

Wilcoxon’s signed rank tests revealed no significant differences between the retest with no mask or a surgical mask (AVQI: *p* = 0.704, ABI: *p* = 0.873) nor significant differences between the retest with no mask or an FFP-2 mask (AVQI: *p* = 0.626, ABI: *p* = 0.703).

## Discussion

No proband or patient can read a passage twice exactly the same. Thus, repeated measurements will lead to slightly differing results. Barsties&Maryn proposed a value of 0,54 for the absolute retest difference of AVQI-values, which is very similar to what we found (Table 2). Their paper did unfortunately not present ABI values [16] and the authors of this work are not aware of any other such data for the ABI in German.

In our study, the mean absolute AVQI difference between two readings with no mask was 0.45 (standard error 0.051). If the surgical mask impeded the measurement one would expect differences to the base measurement larger than that. They were however very close with 0.48 (standard error 0.071) and so was the mean of the differences to the FFP-2 mask at 0.51 (standard error 0.074). For all three conditions, the mean difference in repeated measurements was around 0.5 and thus very good for a measurement range from 0 to 10. The standard errors are sufficiently small to state that the presence or absence of a surgical or FFP-2 mask did not constitute a clinically relevant difference. Essentially the same is true for the ABI.

Surgical masks not making a clinically relevant difference was to be expected since earlier studies have shown little or no influence of surgical masks on a large number of single electroacoustic measures and AVQI and ABI are just combinations of such electroacoustic measures. We consider it still worthwhile to have tested the specific combination of measures performed in this procedure.

One limitation of our study is the sample size. Other studies investigated the influence of gender [5] or age groups [7]. Goyajev et al. reported profession and smoking, albeit without evaluating it [8]. That data is not available for our study. More important, as with most publications, we did not include dysphonic voices and it is very well possible, that mask-induced alterations of the voice are more prominent dysphonic patients. As a last limitation mentioned here, it remains unclear how much this can be generalized to masks of different manufacturers. The authors are confident because earlier studies that showed no important impact of masks on electroacoustic voice measures have presumably used different masks than us and because if different masks altered people’s voice much more that would be detectable by ear and harm the sales of such masks.

## Conclusion

AVQI and ABI are measures of voice quality that can be measured with or without surgical or FFP-2 masks and comparisons of measurements with and without masks are valid. AVQI/ABI thus lend themselves to voice diagnostics in times when loud utterances and utterances without a mask may put the therapist or other patients at air-borne infection risk.

## Data Availability

Raw data are available as electronic supplementary data to this publication.
